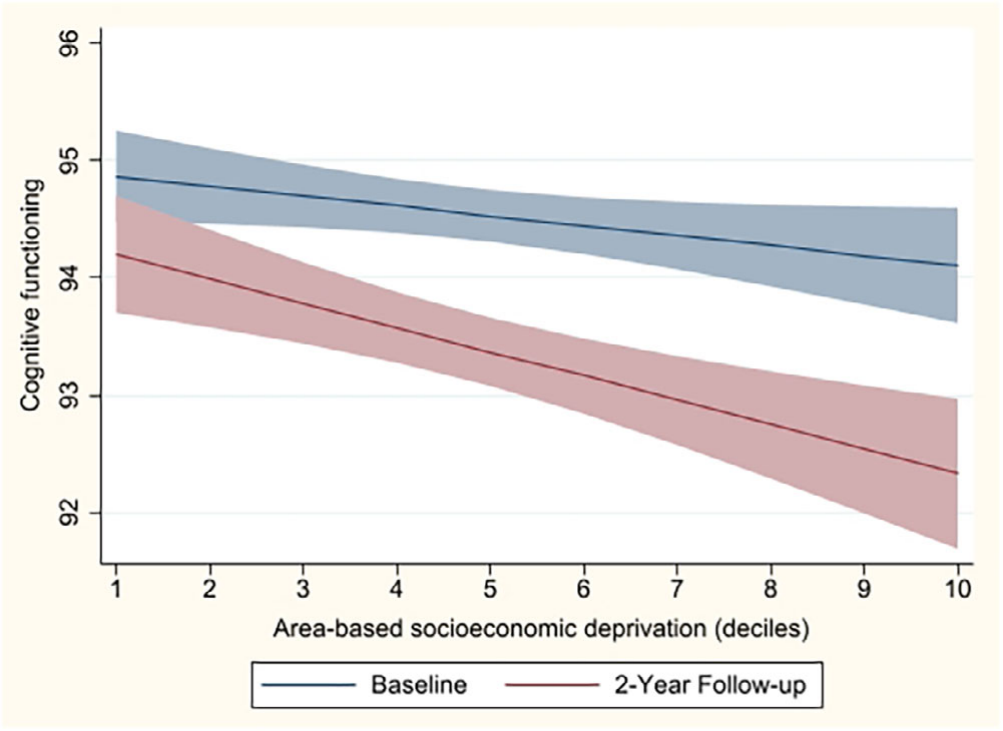# Area‐based socioeconomic deprivation is associated with cognitive decline in midlife to early late‐life New Zealanders without cognitive impairment

**DOI:** 10.1002/alz.089779

**Published:** 2025-01-09

**Authors:** Susanne Röhr, Rosemary Gibson, Fiona Alpass

**Affiliations:** ^1^ School of Psychology, Massey University, Auckland, Auckland New Zealand; ^2^ Global Brain Health Institute (GBHI), Trinity College Dublin, Dublin Ireland; ^3^ School of Psychology, Massey University, Palmerston North, Manawatu New Zealand

## Abstract

**Background:**

Research identified individual‐level socioeconomic factors as key determinants of cognitive health. This study investigated the effect of area‐based socioeconomic deprivation on cognitive outcomes in midlife to early late‐life New Zealanders without cognitive impairment. Understanding geographical dimensions of socioeconomic determinants of cognitive health is important from an equity perspective.

**Method:**

Data stemmed from a subsample of the New Zealand Health, Work and Retirement Study, a cohort study on ageing. In 2010, 1,001 participants aged 49‐84 years completed face‐to‐face interviews and were reassessed two years later. Cognitive functioning was measured using Addenbrooke’s Cognitive Examination–Revised, adapted for culturally acceptable use in New Zealand. Area‐based socioeconomic deprivation was assessed using the New Zealand Deprivation Index (NZDep2006). Linear mixed‐effects models analysed the association between area‐based socioeconomic deprivation and cognitive outcomes, controlling for individual‐level socioeconomic (age, age², gender, education, ethnicity [Māori, Indigenous people of New Zealand, and Non‐Māori, mostly of European descent], marital status, employment, net personal income), lifestyle and health variables (Lifestyle for Brain Health/LIBRA index, social loneliness).

**Result:**

The analysis included 783 participants (54.7% female, mean age 62.7 years, 25.0% Māori). Individuals with cognitive impairment at baseline (n = 69) and older than 75 years were excluded (n = 79). Further attrition was due to missing data. At baseline, 39.7% resided in low deprivation areas, 39.0% in moderate, and 21.3% in high deprivation areas. The unadjusted model indicated a significant association between higher area‐based socioeconomic deprivation and lower cognitive functioning (B = ‐0.16, 95%CI: ‐0.22,‐0.10; *p* < .001) and cognitive decline (B = ‐0.12, 95%CI: ‐0.21;‐0.03; *p* = .015). The adjusted model yielded similar results for cognitive functioning (B = ‐0.08, 95%CI: ‐0.15;‐0.01; *p* = .050) and cognitive decline (B = ‐0.12, 95%CI: ‐0.20;‐0.04, *p* = .013) (Fig. 1). Influential covariates included gender, education, and lifestyle (LIBRA).

**Conclusion:**

This study demonstrated a relationship between higher area‐based socioeconomic deprivation and lower cognitive functioning, along with cognitive decline, in cognitively unimpaired New Zealanders aged 48 to 75 years. These findings emphasize the importance of considering neighbourhood characteristics and broader socioeconomic factors in strategies aimed at mitigating cognitive health disparities and reducing the impact of dementia in disadvantaged communities.